# Overstating the effects of anthropogenic climate change? A critical assessment of attribution methods in climate science

**DOI:** 10.1007/s13194-023-00516-x

**Published:** 2023-03-11

**Authors:** Laura García-Portela, Douglas Maraun

**Affiliations:** 1 Institute for Technology Assessment and System Analysis, Karlsruhe Institute of Technology, Karlsruhe, Germany; 2 Environmental Science and Humanities Institute, Department of Geosciences, University of Fribourg, Chemin du museé 4, 1700 Fribourg, Switzerland; 3 Wegener Center for Climate and Global Warming, University of Graz, Brandhofgasse 5, 8010 Graz, Austria

**Keywords:** Extreme weather events, Probabilistic approach, Storyline approach, Overstatements of anthropogenic climate change

## Abstract

Climate scientists have proposed two methods to link extreme weather events and anthropogenic climate forcing: the probabilistic and the storyline approach. Proponents of the first approach have raised the criticism that the storyline approach could be overstating the role of anthropogenic climate change. This issue has important implications because, in certain contexts, decision-makers might seek to avoid information that overstates the effects of anthropogenic climate change. In this paper, we explore two research questions. First, whether and to what extent the storyline approach overstates the effects of anthropogenic climate change. Second, whether the objections offered against the storyline approach constitute good reasons to prefer the probabilistic approach. Concerning the first question, we show that the storyline approach does not necessarily overstate the effects of climate change, and particularly not for the reasons offered by proponents of the probabilistic approach. Concerning the second question, we show, independently, that the probabilistic approach faces the same or very similar objections to those raised against the storyline approach due to the lack of robustness of climate models and the way events are commonly defined when applying the probabilistic approach. These results suggest that these objections might not constitute good reasons to prefer the probabilistic approach over the storyline approach.

## Introduction

We know that anthropogenic greenhouse gas emissions and other human activities are a major forcing of recent climatic changes. We are less certain about the link between particular extreme weather events (EWEs) and anthropogenic forcing since EWEs would occur even in a preindustrial climate (IPCC, 2021, AR6, Ch 11, Sect. 2.3 and 2.4 ). However, this link seems to be of particular societal interest because changes in local weather affect societies more directly (Allen, 2012; Stott et al. 2016; Nature Editorial, 2018). For instance, some have claimed that attribution studies are relevant for adaptation measures because ‘based on the occurrence of a particularly damaging extreme event, plans could be made to adapt to an increasing frequency of such events in future’ (Stott et al., 2016, 24; similarly in Stott et al., 2017); or for advancing justice-claims, such as these related to compensation for loss and damage (Thompson & Otto, 2015). Moreover, claims concerning compensation for damage from anthropogenic climate change (ACC) are reaching courts and gaining attention in climate litigation (Burger et al., 2020).^1^

Scientists have contributed to these pressing social demands by proposing different attribution methodologies to establish a link between EWEs and ACC. The first methodology to emerge was the probabilistic approach – also known as risk-based approach, probabilistic event attribution or just PEA (Allen, 2003; Stott et al., 2004, 2013, 2016; Otto et al. 2017).^2^ Later, another group of scientists offered an alternative based on conditional attribution (Trenberth et al., 2015), which is a specific application of a specific type of storyline approach (Shepherd, 2016; Shepherd et al., 2018). In line with other publications in the philosophy of science literature, we refer to this approach as the storyline approach.

Although proponents of the storyline approach have always attributed a complementary role to their approach, their proposal generated some controversy among the PEA scientific community. The main objection has been that the storyline approach could be overstating the role of ACC in EWEs (Allen, 2011; Stott et al., 2013, 2016, 2017). In fact, the controversy has led to a general association of the storyline approach with overstatements of ACC, in contrast to the probabilistic approach.

Importantly, this categorization might influence decision-makers. Those stakeholders who seek to avoid overstating the effects of ACC might base their decision-making on the results provided by PEA studies instead of studies carried out with the storyline approach. For instance, this categorization might be relevant in liability contexts. If stakeholders seek to avoid being partial for or against one of the parties, they might forgo using an attribution method that presumably overstates the effects of ACC because such a method might be partial against the putative liable parties. Similarly, if the decision is about directing adaptation funds to a particular region or investing these funds on development aid in a different one, they might want to avoid methods that overstate the effects of ACC. After all, the results of such a method would presumably make the adaptation problem look worse than it is and therefore generate some biases towards the adaptation project. These might constitute reasons to prefer an alternative approach that presumably would not overstate the effects of ACC.

Hence, we believe that the association between the use of the storyline approach and overstatements of ACC, and how this association affects the choice between different attribution methods, deserves further clarification and investigation. In this paper, we explore two research questions. First, whether the storyline approach necessarily overstates the effects of anthropogenic climate change. Second, whether the objections offered against the storyline approach constitute good reasons to prefer the probabilistic approach. In a nutshell, we argue that the storyline approach does not necessarily overstate the effects of ACC and that the objections offered against this approach cannot constitute good reasons to prefer, in general, the probabilistic approach because this one is often affected by the same or very similar objections.

We proceed as follows. In Section 2, we explain the differences between the probabilistic approach and the storyline approach. In Section 3, we briefly describe the reaction of the PEA community and the scientific controversy generated with the development of the storyline approach. In Section 4, we argue that, in many cases, the storyline approach does not overstate the effects of ACC for the reasons offered by the PEA community. In Section 5, we provide an independent argument showing that, in fact, the probabilistic approach is vulnerable to the criticisms raised against the storyline approach because of the lack of robustness of climate models, the way EWEs are commonly defined when applying the probabilistic approach and the dominance of thermodynamic changes over dynamic ones. These results suggest that these objections might not constitute good reasons to prefer the probabilistic approach over the storyline approach.

## Attribution methods: the probabilistic and the storyline approach

Generally, attribution science aims at identifying in which sense and/or to what extent a certain EWE can be attributed to ACC. However, this general question can be interpreted in at least two different ways. The probabilistic approach and the storyline approach differ precisely in the way they approach that general question.

The probabilistic approach has so far been the conventional methodology in attributing EWEs to human forcing (Allen, 2003, 2011; Stott et al., 2013, 2016; Mera et al., 2015; Otto et al., 2017). This methodology takes a certain event as a token of a class of EWEs and asks the following research question: How much did ACC increase the probability or risk of a specific type of event? Answering this question requires comparing the probability (p1) or risk of a specific class of events in a world affected by ACC (actual world) and the probability (p0) of such a type of event in a world without ACC (counterfactual world).

The result of this process would be statements of this sort: ‘ACC has increased the probability of occurrence of this type of EWE by a factor X (probability ratio)’. Or, put in other words: ‘an event of that class was X% more likely to occur in a world with ACC than in a world without ACC’. This operation is often represented as a Fraction of Attributable Risk (FAR), where FAR = 1 – (p0/p1). The FAR is interpreted as the fraction of the risk of an event that is attributable to the external forcing. Hence, FAR leads to probabilistic causal claims, such as: ‘it is very likely that X amount of the risk of this EWE is attributable to anthropogenic forcing’. In sum, the probabilistic approach attributes a fraction of the probability or the risk of the event occurring to ACC.

Of course, it is also possible that this methodology shows a decrease in the probability of the EWE occurring due to ACC. Therefore, statements of the sort ‘ACC has decreased the probability of occurrence of this type of EWE by a factor X’ or ‘an event of that class was X% less likely to occur in a world with ACC than in a world without ACC’ are also possible. Although scientists working within this approach are less prone to emphasize this point, it is important to bear in mind that the influence of ACC on the EWE can also work in the opposite direction and decrease the probability of the event occurring. It is also possible that the probability of the event is unaffected by ACC and remains essentially the same.

Importantly, the PEA community has suggested that the results of their studies could support the attribution of the harmful effects of EWEs to ACC when certain thresholds are reached, as is done in tort law contexts (Stott et al., 2004; Allen et al., 2007; Allen, 2012; Thompson & Otto, 2015; Otto et al., 2016). In tort law contexts, factor X can be said to have caused an effect Y if, on the balance of probabilities, it is more likely than not that X caused Y (Lloyd et al., 2021). For this to be the case, X must have more than doubled the probability of Y occurring. Famously, this threshold has been successfully applied in asbestos lawsuits, where certain negative health conditions were attributed to the use of asbestos because it was considered that this substance more than doubled the probability of their occurrence. With this in mind, many scholars have suggested that a 0.5 FAR could be considered a relevant threshold for attributing the harmful effects of EWEs to ACC, which can be relevant in liability contexts (Grossman, 2003; Pall et al., 2011; Allen, 2012; Hannart et al., 2016; Stuart-Smith et al., 2021).^3^

The application of the storyline approach to attribution studies emerges from certain skepticism concerning the application of probability assessments to a certain type of EWE.^4^ To explain this skepticism, proponents of the storyline approach point out the different contributions of dynamic and thermodynamic climate variables to an EWE (Trenberth et al., 2015; Shepherd et al., 2018). Simplifying, dynamic factors include specific weather patterns such as cyclonic storms or persistent blocking highs that are responsible for the *occurrence* of a given weather event at a given time. Thermodynamic factors include, e.g., surface warming and moistening of the atmosphere, and strongly influence the *severity* of an EWE.

For the thermodynamic aspect of the event, models typically simulate robust changes in a warming climate, but changes in atmospheric dynamics are usually much more uncertain. Indeed, Shepherd affirms that: ‘the most uncertain aspect of climate modeling lies in the representation of unresolved (sub-gridscale) processes such as clouds, convection, and boundary-layer and gravity-wave drag, and its sensitive interaction with large-scale dynamics. It is, therefore, reasonable to hypothesize that the representation of these processes is responsible for systematic non-robustness of the predicted circulation response to climate change’ (Shepherd, 2014, 706). For these reasons, defenders of the storyline approach argue that identifying a possible human contribution to changes in dynamic climate variables is very challenging and it often delivers unreliable or inconclusive results. Similarly, Trenberth et al. argue that ‘although large changes in atmospheric circulation can be readily apparent in a single climate model run, they are not robust and can change considerably in the next run or model’, and, importantly, that ‘forced circulation changes are not well established, and it is difficult to detect changes in circulation-related extremes in observations because of small signal-to-noise ratios’ (Trenberth et al., 2015, 725).

The problem is that, because the PEA community wants to consider the EWE as a ‘single, self-reinforcing and indivisible whole’ (Allen, 2012, 13), the probabilistic approach aims to track both dynamic and thermodynamic changes in the EWE due to ACC. But this would be problematic, according to proponents of this approach, because of the cited challenges in representing dynamic changes. Hence, these scientists conclude that ‘the conventional approach to extreme event attribution [PEA] is rather inefficient in cases that are strongly governed by changed circulation, with a generally inconclusive outcome. Even when a detectable anthropogenic influence is found in a model, the reliability of that finding cannot carry much weight’ (Trenberth et al., 2015, 726). In a nutshell, the criticism is that these problems might make attribution studies miss the effects of ACC and also undermine their reliability.^5^

However, scientists working on the storyline approach do not refuse to attribute EWE (or, at least, some aspects of these events) to anthropogenic forcings.^6^ Instead of focusing on types of EWE, the storyline approach focuses on concrete events, and investigates their sources in a conditional manner (Shepherd, 2014, 2016). Shepherd has described the storyline approach as ‘analogous to accident investigation (where multiple contributing factors are generally involved and their roles are assessed in a conditional manner)’ (Shepherd, 2016, 32). To do so, scientists proceed by taking the large-scale dynamic state of an event as a given constraint and then ask about the contribution of human forcing to the event’s thermodynamic climate variables. In that way, they obtain answers to the attribution question conditioned on the given dynamic components (see, for instance, Pall et al., 2017; Patricola & Wehner, 2018; Takayabu et al., 2015; Sillmann et al., 2021; IPCC AR6 Ch 11, Sect. 11.2.3). This approach is less prone to errors related to the unreliability of climate models because it does not depend on the ability of these models to simulate changes in atmospheric circulation.

After fixing the dynamic variables, the storyline approach shifts the research question into one about the event’s magnitude or severity. Instead of asking how much anthropogenic forcing has increased the likelihood of the event happening, this approach focuses on the effects of anthropogenic forcing in the increase of the event’s magnitude. That is, the relevant research question is not: ‘how much has anthropogenic forcing increased the likelihood of the event happening?’. Instead, the question is: ‘how much has ACC increased the magnitude of this particular event?’. Accordingly, the answers attached to this methodology are of this sort: ‘ACC increased the EWE’s magnitude by a value or factor of X’. As before, the methodology can also show a decrease in the magnitude, thereby leading to statements such as: ‘ACC has decreased the EWE’s magnitude by a value or factor of X’.

## The reaction and criticism of the PEA community towards the storyline approach

The emergence of the storyline approach triggered some controversy within the scientific community, and especially among the PEA community. Their main complaint is that the conditional structure of the storyline approach and its focus on thermodynamics could make this approach overstate the effects of ACC.

This worry has been captured in various papers from different proponents of the PEA community. For instance, Stott et al., argued that ‘by always finding a role for human-induced effects, attribution assessments that only consider thermodynamics could overstate the role of ACC’ (2016, 33; similarly in Stott et al., 2017, 147). Similarly, Allen (2011) accused some proponents of the storyline approach – in particular, (Trenberth, 2011; Curry, 2011) – of assuming that ACC had always an impact on local weather events and that this puts scientists at risk of making false-positive errors. The spirit of this complaint was largely in line with that of Stott and colleagues, namely, that the new alternative approach could be overstating the effects of ACC.^7^ These complaints also imply that the probabilistic approach might be less prone to overstate the effects of climate change and, if anything, it has the opposite tendency, thereby position it more in line with values of scientific rigour.^8^

Some philosophers of science have interpreted this criticism as suggesting a tendency of the storyline approach towards overstatements. Accordingly, the criticism of the PEA community would be that the storyline approach is *prone* to overstating the effects of ACC (Lloyd & Oreskes, 2018, 2019; Winsberg et al., 2020; Pulkkinen et al., 2022). However, the PEA community has neither shown the existence of such a trend, nor have they expressed their criticisms in these terms, but rather in vaguer ones. Their explicit formulation is that this approach ‘could’ overstate the effects of ACC because of its focus on thermodynamic variables. This merely refers to a possibility but not to a general trend, as interpreted by these scholars. However, the formulation of this criticism and the reception of their ideas by the scientific community suggests, at least, the existence of a general association between the use of the storyline approach and overstatements of ACC in the literature, presumably because of the conditional nature of this approach and its focus on thermodynamic variables.

This paper offers arguments that cast doubt on this general association, leaving aside the question of whether the storyline approach is prone or not to overstatements. The examples below aim to highlight cases where this general association might not hold. With this point, we take ourselves to at least show that the conditional nature of the storyline approach does not necessarily lead to overstatements of ACC. We are aware that our arguments here do not disprove statements about general trends (i.e., they do not show that the storyline approach does not overstate the effects of anthropogenic climate change) and that they do not show the opposite trend (i.e., that the storyline approach understates the effects of ACC).^9^ Nonetheless, we believe that our discussion provides two important contributions in this regard. First, that if the storyline approach were prone to overstate the effects of ACC, then it would be so for reasons other than those provided by PEA proponents (i.e., not *simply* because of the conditional nature of the approach). Second, that the storyline approach can also understate the effects of ACC and they also need to be taken into consideration if one aims to analyze the proness of each method towards over- or understatements.

Finally, for clarity’s sake, we lay out two plausible interpretations of the complaint that the storyline approach overstates the effects of ACC, which differ mostly in their respective focus.^10^ First, the criticism could focus exclusively on weather event itself. According to this interpretation, the complaint of the PEA community against the storyline approach would be that the storyline approach states that ACC has increased the magnitude of the EWE more than it has in fact done it. For example, here the complaint could be that the storyline approach affirms that ACC caused extreme temperature happening in a particular geographical location at a particular time to be X degrees higher than expected from natural variability, when in fact ACC only caused that extreme temperature to be X-Y (Y > 0) degrees higher. We call this interpretation of the criticism of overstatement ‘O1’.

Second, the criticism could be focused on the attribution of certain harmful effects or impacts to ACC. According to this interpretation, the complaint of the PEA community would be that the storyline approach often suggests that certain negative impacts are attributable to ACC, when in fact they should not be attributed to ACC. We call this interpretation of the criticism of overstatement ‘O2’. The concern that the storyline approach overstates the effects of ACC in this sense appears implicitly when the PEA community warns against the danger of overadaptation triggered by the results of storyline studies. The concern of the PEA community is that the storyline approach might exaggerate the impacts occurring in particular location due to ACC, thereby suggesting investing money to adapt to the negative effects of ACC where in fact is not needed (Stott et al., 2013, 2016, 2017).

Note that these two interpretations of the PEA community’s criticism are not mutually exclusive, but rather closely related. In fact, they are often implied together in the criticisms of overstatements raised by the PEA community against the storyline approach. Here, we distinguish them for analytic purposes and highlight that they differ mostly in their respective focus: whereas O1 focuses on the link between changes in climatic conditions and ACC, O2 focuses on the attribution of negative impacts to ACC when certain thresholds are overshot.^11^ Depending on the context of the discussion, one or the other focus might be more relevant to reflect and understand the criticisms of the PEA community.

In the next section, we argue that the storyline approach does not necessarily overstate the effects of ACC for the reasons offered by the PEA community. Later, in Section 5, we move on to provide an independent argument showing that the probabilistic approach is vulnerable to similar criticisms raised against the storyline approach.

## The storyline approach and the criticism of overstatement

Let us start with O1. The complaint here would be that the storyline approach overstates the effects of ACC because it affirms too much of an increase in the magnitude of an EWE due to ACC. The complaint by the PEA community is based on the conditional nature of the storyline approach, that is, on the fact that the storyline approach focuses only on the thermodynamics and take the dynamic variables as fixed. The combination of two factors might support the belief that the storyline approach would typically show an increase in the magnitude of an EWE. These factors are the well-reported general increase in global temperature due to climate change and the focus of the storyline approach on thermodynamic climate variables. The idea could be that because of the robust connection between increases in global temperature and ACC, an approach that is focused on thermodynamic changes (strongly related to temperature changes) would typically find increases in the magnitude of the event due to ACC.

Note that this complaint assumes that the dynamics of the atmosphere that are taken as a given for a certain EWE make the results of the storyline approach affirm that ACC had increased the magnitude of the event more than if we could account for dynamic changes caused by ACC, which, in fact, contributed to the EWE. Or, in other words, that complaint assumes that if we could reliably account for the actual changes in the dynamics due to ACC (which often remain uncertain), we would see that ACC made the event less severe than the storyline approach shows. Scientists working with the probabilistic approach have emphasized this point by offering case studies where detectable dynamic changes reduce the effects of ACC in comparison with these shown by only taking thermodynamic changes into consideration (Otto, 2015; Otto et al., 2016; Pall et al., 2011). For instance, Otto et al. (2016), refer to the heavy flooding in Germany in 2013, where some parts of the southeast region received a month’s worth of precipitation in 3 spring days (Schaller et al., 2016). As they argue, we would expect the likelihood of this event to increase with ACC because the vapor capacity of the atmosphere increases with warming. In fact, the study shows that an increase of 0.9 K of temperature in the region and season would increase the likelihood of such rains by approx. 6%. Such an increase would render a 1-in-200-year event in a preindustrial climate a 1-in-120-year event with ACC. However, simulations of the overall change in risk show no change in the likelihood of the event occurring. This result implies, as Otto et al. conclude, that there is an important role of atmospheric circulation in counteracting the increase in probability that would be expected by only considering thermodynamic factors (Otto et al., 2016, 815).

However, this concern ignores the fact that actual changes in the dynamics could also make an EWE more severe than shown by only considering thermodynamic changes caused by ACC. In fact, cases of this sort can also be found in the literature, where detectable dynamic changes show the opposite effect, that is, an increase in the detected effects of ACC in comparison to these detected when only tracking thermodynamic changes (Schaller et al., 2016; Pfahl et al., 2017). In these cases, a conditional approach – such as the one used in most storyline approach studies – understates the effects of ACC because its results would only be based on thermodynamic changes. Thus, in a nutshell, the fact that the storyline approach fixes the dynamic variables and focuses on the thermodynamics does not necessarily make this approach overstate increases in the magnitude of EWEs. Hence, it is not true that by focusing only on the thermodynamics, the storyline approach necessarily overstates the effects of ACC according to O1.

Let us now turn to O2. To recall, according to this interpretation, the complaint of the PEA community would be that the storyline approach suggests that ACC has caused (or will cause) certain harmful effects when in fact it has not. Following the PEA community’s concerns, we want to explore whether the storyline approach necessarily overstates the effects of ACC in this sense, thereby suggesting, for instance, to invest adaptation funds in regions that are in fact not affected by climate change (not at least to a significant degree), or to make polluters liable for the harmful effects of ACC in a certain region.

First, it is worth recalling that studies conducted with the storyline approach do not only report increases but also decreases in the magnitude of EWE (see Section 2). Since most harmful impacts are caused by increases in the magnitude of EWE, the storyline approach would not suggest that ACC has caused certain negative effects when reporting decreases in the magnitude of certain EWE. This remark rejects the idea that the storyline approach always implies that ACC has an impact on harmful effects caused by local weather events, as implied by Allen (2011).

Nevertheless, here, the combination of the following factors might again support the belief that the storyline approach would typically suggest that the harmful impacts associated with an EWE have been caused by ACC, or at least that ACC has significantly contributed to causing those impacts. As before, two factors are the well-reported general increase in global temperature due to climate change and the focus of the storyline approach on thermodynamic climate variables. The third one is the positive effect of temperature increases on many hazards (hotter heatwaves, more intense rain, etc.). The idea here would be that because of the robust connection between increases in global temperature and ACC and the connection between temperature increases and many hazards, an approach that is focused on thermodynamic changes (strongly related to temperature changes) would identify increases in the magnitude of a local event due to ACC and thus a connection between ACC and certain harmful impacts. This again might suggest that the storyline approach always finds that ACC has caused or at least has had a significant contribution to the occurrence of harmful impacts, thereby overstating the effects of ACC.

Second, however, as mentioned above, recall that accounting for the effects of ACC in dynamic changes could also show that in fact the EWE was made more severe than shown by only considering thermodynamic changes caused by ACC (i.e., with storyline approach studies). In this case, only considering thermodynamic changes would underestimate the magnitude of the impacts attributable to ACC. Accordingly, then, storyline approach studies could also be underestimating the harmful effects caused by ACC.

Third, and perhaps most importantly, the storyline approach could be combined with a decision threshold to limit the attribution of harmful impacts to ACC, similar to the one suggested by some PEA scholars. Recall that PEA studies do not attribute all EWE to ACC for which some increase in probability is detected. Instead, scholars working with the probabilistic approach have suggested that PEA studies could use a threshold (usually, 0.5 FAR) of increased likelihood to attribute EWEs to ACC. The idea is that when this threshold is exceeded, following the standards of certain legal contexts, one could attribute the EWE to ACC. Often, PEA scientist imply that this procedure could not only be used to attribute the EWE themselves, but also their harmful impacts to ACC. This is made especially clear when suggesting that this procedure could be used in liability and compensatory contexts (Allen, 2003, 2012; Allen & Lord, 2004; Allen et al., 2007; Thompson & Otto, 2015). One cannot seek compensation or be made liable for the occurrence of extreme rainfall precipitation or for extreme temperatures alone, but rather for their negative impacts on their property or their health. Hence, the claim that attribution studies linking EWE to ACC could be used in liability and compensatory contexts seems to assume that the decision threshold of 0.5 FAR does not only serve the purposes of attributing the EWE to ACC but also the harmful impacts resulting from that EWE. That is, the 0.5 FAR seems to work implicitly as a threshold to attribute certain negative impacts to climate change, even if the selection of that threshold is only justified on legal grounds. This threshold is not intrinsic to PEA, but it is rather an addition to decide which impacts are attributable to ACC and which not, for legal, political, economic or other societal purposes. Notably, this threshold implies that some of the effects of ACC are left aside or ignored. For instance, the use of this threshold would exclude the attribution of any climate change-related impacts for those EWE for which a probability increase is positive but less than 100% (i.e., 0.5 FAR), even if the probability of the event has been increased by 99% due to ACC.

Our point here is that *if* something like this is acceptable for the probabilistic approach, the results of the storyline approach could also be combined with a threshold to decide whether the impacts associated with a certain EWE are so relevant as to be attributed to ACC for similar legal, political, economic or other kinds of societal purposes. For instance, imagine that we want to assess whether certain impacts (for instance, a flood, or the property losses derived thereof) associated with an EWE (for instance, heavy rainfall) can be attributable to ACC, perhaps for deriving compensatory claims. In this case, one would not refer to a ratio of conditional probability, as in PEA studies. But, instead, one could use an absolute meteorological threshold to derive impact attribution claims, which would exclude some of the results derived from storyline approach studies. That is, one might artificially use this threshold to derive attribution claims for the impacts of EWE that reach a certain magnitude and exclude those that do not reach this magnitude. Such a relevant meteorological threshold for the storyline approach could be derived from expert knowledge or from impact model studies assessing the sensitivity of an impact to climate change.^12^ For instance, in case of a flood caused by heavy precipitation, the meteorological threshold could be informed by a relevant increase in runoff or by a relevant increase of the damage to some critical infrastructure. Also, a threshold could be defined relative to natural fluctuations of an impact, e.g., typical variations in runoff. If the identified effect would exceed a chosen multiple of these fluctuations (e.g. 2σ), this effect would be attributed to ACC. Such a threshold would be conceptually similar as the PEA threshold (both compare a signal with natural fluctuations), but it would not be identical in the sense that it would not necessarily issue the same attribution statements as the PEA approach. In any case, the point here is not to provide a unique approach to define such a threshold, but to highlight that different possible avenues could be used to define a threshold for attribution statements that can serve different purposes.

Even if we believed that the focus of the storyline approach on thermodynamic changes tends to overstate the effects of ACC in the sense O1 (that is, by overstating the magnitude of the weather event itself), a decision threshold similar to the one implied or suggested by some PEA scientists could prevent, or at least limit, overstatements in the sense O2 when using the storyline approach (that is, overstatements of impacts due to ACC).^13^

We would like to highlight two important points here. First, this decision threshold would be an artificial addition to the results obtained from the storyline approach, but not part of the storyline studies themselves. But this should not be a reason to reject this possibility *if* one accepts the relevance of this (similar) procedure for PEA studies. Second, there is no reason to believe that this procedure would overstate the effects of ACC in the sense of overstating its harmful impacts (overstatements in sense O2) if the storyline approach is employed more than if the probabilistic approach is employed. In fact, whether this kind of impact attribution would overstate the effects of ACC would depend on how thresholds are set for deciding when certain impacts are attributable to ACC for different societal purposes. This last remark should not be surprising since, as suggested by Allen and his colleagues, causal attribution claims are not only scientific issues (Allen et al., 2007, 1354). Scientific research needs to be combined with a certain understanding of causation and on a certain understanding of which thresholds are relevant to claim causation in different contexts and with different purposes.

## On how the probabilistic approach is affected by similar objections

In this section, we provide an independent argument for why the PEA criticisms do not constitute good reasons to prefer the probabilistic approach over the storyline approach. We argue that, very often, the probabilistic approach faces similar objections to those raised by the PEA community against the storyline approach. This is due to the lack of robustness of climate model simulated changes, the way events are commonly defined when applying the probabilistic approach and the stronger signal of thermodynamic changes over dynamic ones. In our view, the fact that the probabilistic approach faces similar criticisms undermines a general preference for the probabilistic approach over the alternative one, independently of the success of our previous arguments.

Let us start with the role of lack of robustness of simulated dynamic changes in climate models. As we saw, climate model results are very uncertain with respect to changes in dynamic factors. For several cases and regions, different climate models even simulate opposite *changes* in aspects related to the atmospheric circulation (Doblas-Reyes et al., 2021; Zappa et al., 2021) such as changes in European wind speed (Zappa & Shepherd, 2017) or in central European precipitation patterns (Maraun, 2013). A plausible uncertainty range can thus only be derived by an ensemble of multiple climate models that spans all plausible changes in the atmospheric circulation. State-of-the-art multi-model ensembles typically comprise some 10 to 30 different (although not independent, as they may share several components) climate models (e.g., Eyring et al., 2016; Jacob et al., 2016). But because of the high computational costs of simulating climatic changes under different forcings, many classical event attribution studies have been conducted based on a single model only (e.g., Stott et al., 2004; Pall et al., 2011; Lott et al., 2013). The world weather attribution (WWA) initiative demands ‘at least two and preferably more models to be good enough for the attribution analysis.’(WWA, 2021). But selecting only a few climate models to represent changes in the atmospheric circulation and related phenomena can cause substantially misleading conclusions, including overstatements of the role of anthropogenic forcing.

Imagine the attribution of a heavy rainfall event in the presence of strong dynamic uncertainties. Some models may, for the considered region, simulate an increase in heavy precipitation under ACC, some a negligible change, and some a decrease. Models from the first group would suggest an increase in the likelihood and/or FAR, those from the second group an essentially constant value, and those from the third group a decrease in the likelihood and/or FAR. Given the uncertainties in dynamic changes, the true change under ACC is not known and may be in either group (or even outside, if uncertainties are not reliably sampled because of common model errors).

Selecting only a small number of models increases the danger of missing one of the groups, thereby missing the true climate change signal, and ultimately of producing an overstatement.^14^ If the true effect of ACC would be an increase in the occurrence probability of the event, but the selected ensemble would only include models showing no or a negative change, the influence of ACC would be understated. But vice versa, if the true effect of ACC would be a decrease in the occurrence probability, but the ensemble would include only models showing no or positive changes, the influence of ACC would be overstated.

The problem is aggravated by the criteria recommended for selecting suitable models for event attribution (Mitchell et al., 2017; WWA, 2021; van Oldenborgh et al., 2021): they are all based on the performance at reproducing key aspects of extreme events in the *present climate*, but this does not ensure a credible representation of *changes* in extreme events. Let us assume that the spread in climate change signals across the full ensemble would represent the true uncertainty we have about climate change.^15^ Selecting only a subset of these models – and thus reducing ensemble spread – without giving any physical argument of why this sub-sampling should reduce the true uncertainty (i.e., with an argument that links present-day model performance to the credibility of the climate change signal) would thus in general underestimate the true uncertainties. To fully represent uncertainties, an additional model selection criterion regarding the representation of model spread is thus required, but this is not included among the listed criteria.^16^ In any case, the key point is: if only a small number of models is considered, the probabilistic approach can, depending on the case and model choice, yield overstatements of the role of ACC.^17^

Let us now turn to the role of defining the event under consideration. Recall the PEA complaint that the storyline approach overstates the effects of ACC by only considering the thermodynamics. Presumably, the PEA community takes this to be a reason to favour the probabilistic approach because, in theory, this approach considers both the dynamic and thermodynamic variables. However, as we will show, the way events are commonly defined in PEA studies focus very often only on the thermodynamic variables of the EWE, leaving aside the dynamic ones.

Recall that the probabilistic approach asks about the occurrence of type of events. For conducting their counterfactual analysis, PEA studies must define the kind of event they are interested in with some level of abstraction. In doing so, PEA studies operate with a simple, one-dimensional definition of the event. This simple definition leaves aside some of the atmospheric, meteorological factors and temporal aspects characterizing the particular event that motivated the attribution question, which is essentially multi-dimensional. We call the one-dimensional definitions ‘proxy-definitions’, as they constitute only a simple approximation to the set of atmospheric and meteorological conditions characterizing the particular event that led to impacts raising the public’s interest.

Our point here is that these proxy-definitions are often designed in a way that leaves aside or downplays dynamic factors, as it occurs in studies conducted with the storyline approach. For instance, extreme events such as droughts and heatwaves are often caused by an interplay of dynamic and thermodynamic aspects. In the mid-latitudes, dry spells and heatwaves are typically caused by persistent blocking high-pressure systems (Woollings et al., 2018). However, PEA attribution studies typically express these multidimensional events, which have a distinct dynamic aspect, with simple, one-dimensional definitions. For instance, the 2003 European heatwave was caused by a blocking high, persistent for several weeks, and amplified by low soil-moisture conditions (Fischer et al., 2007). But, in their attribution study of the 2003 European heatwave, Stott et al. (2004) characterized the event by *the June-August mean temperature*. Similarly, in 2018 Western, Central and Northern Europe were struck by a severe several-month long drought caused by recurring blocking conditions and accompanied by several heatwaves (WMO, 2019). But the World Weather Attribution initiative characterized this event by the *3-day maximum temperature average in 2018* (WWA, 2018). In both event definitions, the dynamic state – recurring persistent blocking – is ignored.

Admittedly, such one-dimensional, proxy-definitions, focused on thermodynamic factors might have several advantages. First, state-of-the-art climate models still have substantial limitations in representing the dynamics underlying such events, in particular the persistence (Weisheimer et al., 2011; Mitchell et al., 2017; Schiemann et al., 2020), and averaging across longer time scales, or selecting a short period helps to navigate these limitations as seasonal means and daily statistics are usually well represented. Second, similarly, pure temperature indices – by definition – have a strong thermodynamic climate change signal, such that dynamic uncertainties and internal variability as discussed above are relatively low (Shepherd, 2014). Third, a one-dimensional event expressed by one number is more manageable within the standard FAR framework.

However, these proxy-definitions do not capture the dynamic aspects of the event. This is important for two reasons. Firstly, although focusing on the thermodynamic aspects of the event might have those advantages, it might lead to interpretations that overstate the effects of ACC in both sense O1 (overstatements focused on the weather event) and O2 (overstatements focused on the impacts).

Consider the example of heatwaves, which include a temporal aspect (dynamics: the persistence of the blocking high) and the actual temperatures (thermodynamics). Climate models simulate a broad range of plausible changes in the frequency of European summer blocking yet with an overall tendency towards fewer events (Davini & D’Andrea, 2020). But all climate models simulate a robust increase in European summer mean temperatures and 3-day maximum temperatures (Gutierrez et al., 2021). Thus, even though the kind of multi-dimensional events that triggered the attribution question (the 2003 heatwave and the 2018 and 2022 droughts, which included a dynamical aspect) could potentially become less frequent in a future climate (because of the tendency towards fewer blocking events and the effects of such events on heatwaves), the simplified, temperature-based version of the events captured with proxy-definitions will become more frequent (because of robust increases in mean summer temperature or 3-day summer temperature).

Similarly, the Central European Summer of 2022 was characterized by high temperatures and low rainfall with far reaching impacts on health, energy, agriculture, and municipal water supply (WWA, 2022). This event had a thermodynamic component (the high temperatures) and a dynamic one (absence of rain).^18^ The WWA attribution study chose summer soil moisture as an indicator to define the drought and found a clear increase in the associated FAR. But this definition highlights again the thermodynamically-driven aspects of the event because reductions in soil moisture in Central Europe, particularly during the summer, are driven by evapotranspiration due to increasing temperatures (Douville at al., 2021). Projected changes in the length of dry periods (i.e., the dynamically-driven element of the event), which are not robust (Gutierrez et al., 2021), are thus left aside in this definition. Again, the chosen indicator focuses on the clear thermodynamic component and downplays the role of dynamics.

Note that this procedure, in itself, does not overstate increases in the frequency of the weather event due to ACC. In fact, it is true that the simplified, temperature-based weather event captured with the proxy-definition will become more frequent because mean summer temperature and 3-day maximum temperature average increase in a world with ACC. But, because the initial attribution question is raised by referring to the particular event, which is multidimensional, and the attribution study is carried out for a one-dimensional event captured by the proxy-definition, this increases the danger of interpreting statements about the proxy event as statements about the multi-dimensional event.^19^ Given that those proxy events most certainly increase in a world with ACC but the multidimensional events do not necessarily do so (as described above), this can easily suggest that the effects of ACC on the EWE (including all its dimensions) are higher than they actually are, thereby leading to overstatements of the frequency of occurrence of certain multi-dimensional weather events.

Furthermore, the results of PEA might suggest or favor the interpretation that the negative impacts occurring in the aftermath of the 2003 European heatwave were only due to high mean summer temperature (thermodynamic factors), when in fact they were caused by the interplay between thermodynamic and dynamic factors. Such an interpretation might overstate the impacts associated with ACC because ACC increases mean summer temperatures but not necessarily the set of atmospheric and meteorological conditions that fully characterized the 2003 event and their associated impacts. For instance, the impacts of the 2003 event were also influenced by the blocking high and low soil-moisture conditions. Ignoring the influence of those conditions, which are more uncertain because they are closely tied to the dynamic aspect of the event, might suggest an overstatement of the impacts associated with ACC. In fact, there are reasons to believe that ACC might decrease the frequency of that set of conditions, given that it decreases blocking events present in the 2003 event. For this reason, the probabilistic approach is not free from objections similar to those raised against the storyline approach, which undermines the preference for the former over the latter on those grounds.

Secondly, and most importantly, with this use of proxy definitions, note that PEA studies operate in a very similar way to the storyline approach. Ironically, by using a temperature-based proxy definition, the probabilistic approach essentially disregards dynamic changes and emphasizes the thermodynamic ones. Arguably, this practice is not much different from the one characterizing the storyline approach (that is, the practice of conditioning on an unchanged atmospheric circulation and focusing on thermodynamic changes).^20^

Note that we do not claim that the use of proxy definitions (focused on the thermodynamics) always and necessarily affects PEA studies. In principle, the same type of problem may arise for the other types of events considered by WWA, in particular when these occur on time scales not well represented by climate models. But we do not delve into this question here on whether and how this argument could be extended to other events. However, we believe that our argument works at least for the events we listed here.

Finally, we would like to add a further way in which PEA studies might focus on thermodynamic changes. This point concerns the stronger signal of thermodynamic changes, which might outweigh the role of dynamic changes. We believe that this point might affect all event types considered by the WWA, but we choose here a flood event as an example to illustrate this point.

Consider the severe flooding in Germany and Belgium in July 2021. A slowly moving cut-off low caused unprecedented amounts of rainfall, corresponding to a 400-year event in present climate (Kreienkamp et al., 2021). According to a PEA study, climate change has increased the occurrence probability of such an event by a factor of 1.2 to 9, i.e. the observed rainfall amounts would have been extremely unlikely without climate change (Kreienkamp et al., 2021). This attribution statement is based on the overall changes in rainfall, which themselves are caused by the combined effect of changes in the occurrence of cut-off lows (dynamics) and in the rainfall intensities within cut-off lows (thermodynamics). To understand why this statement focuses on thermodynamic changes, we need to assess both changes separately.

Although currently there are no analyses of cut-off low changes in climate simulations, it has been suggested to use changes in blocking highs as a proxy for cut-off low changes (Maraun et al., 2022), given that cut-off lows tend to develop along with blocking highs (Nieto et al., 2007). Current generation climate models show a large spread of changes in the number of days with a blocking high due to ACC, ranging from increases to decreases, but with a slight decrease when considering the mean over all models (Davini & D’Andrea, 2020). These uncertainties arise from both model uncertainty and internal climate variability (see Section above; see also Woollings, 2010; Woollings et al., 2018). Transferring this finding to cut-off lows, we have substantial uncertainty about the influence of climate change on their occurrence, but expect a slight decrease. But, as sketched above, despite this uncertainty and the overall decrease in event occurrence, the overall PEA states an increase in the occurrence probability of the observed heavy rainfall.

The most plausible explanation of this seemingly contradictory result is a strong increase in the rainfall intensities given a cut-off low, which outweighs the decrease in event occurrence and the large uncertainties about this decrease. In other words: even though the PEA considers the full attribution including changes in dynamics and thermodynamics, it mostly draws its strength from thermodynamic changes.^21^ This argument could be easily transferred to the other types of events considered by WWA.

To close this section, recall that defenders of the probabilistic approach believe that the practice of ignoring dynamic changes is a weakness of the storyline approach and that, at least implicitly, this constitutes a reason to prefer their own approach. However, if PEA operates in a similar manner, quite straightforwardly, the fact that the storyline approach focuses only on thermodynamic variables cannot be a reason to disregard the storyline approach in favor of the probabilistic approach.^22^

## Conclusion

Attribution science is evolving rapidly, and the emergence of new alternative methods triggers the question of which method to follow when it comes to attributing EWE to ACC. Different variables might be relevant to decide on this matter. Among them, there is the performance of each attribution method in estimating the effects of ACC. If we had reasons to believe that one method overstates the effects of ACC, whereas the other does not, this might give stakeholders reasons to prefer one methodology over the other. Whether, to what extent, and in which sense an attribution method overstates the effects of ACC might have relevant implications for decision making. For that reason, we believe that it is worth investigating the association, suggested by the PEA community, of the storyline approach with overstatements of ACC; and also whether the criticisms offered against the storyline approach constitute good reasons to prefer, in general, the probabilistic approach. In this paper, we have argued that there are reasons against such a general association and that the probabilistic approach faces, at least sometimes, similar objections to those pressed by its proponents against the storyline approach.

First, we have argued that the storyline approach does not always overstate the effects of climate change. In a nutshell, we have argued that the fact that the storyline approach fixes the dynamic variables and focuses on the thermodynamics does not make this approach inherently likely to overstate increases in the magnitude of EWEs because unknown dynamic changes could have also made the EWE more severe than shown by the storyline approach. Moreover, we argued that this approach does not necessarily overstate the harmful impacts that are attributable to ACC because this depends on how certain thresholds are established, as it occurs with the probabilistic approach.

Second, we have shown, independently, that the probabilistic approach faces similar objections to those raised by the PEA community against the storyline approach. The lack of robustness of climate models might, in many circumstances, make the results of the probabilistic approach overstate the effects of ACC, depending on the model selection. Moreover, the use of temperature-based proxy definitions might lead to interpretations of the results provided by PEA studies that might overstate the role of ACC on specific EWEs. Furthermore, and most importantly, proxy-definitions essentially deemphasize the dynamic components, thereby operating in the way the PEA community criticized the storyline approach. Finally, something similar might happen when thermodynamic changes dominate over dynamic ones. Thus, the fact that the probabilistic approach faces similar criticisms does not justify a general preference for this approach over the alternative one, independently of the success of our previous arguments.

## Footnotes

1 For a critical discussion about the social relevance of attribution studies, see Lusk (2017).

2 Although one can often find the term ‘risk-based approach’ in the literature to refer to this approach, we forego the use of this term because it implicitly conveys the misleading idea that other approaches (i.e., the storyline approach) do not provide a risk-assessment.

3 Here two things should be noted. First, exceeding this threshold means that ACC has increased the probability of the event by more than 100% or has more than doubled the risk of the EWE occurring. Second, here that the suggestion of using the FAR value to derive attribution claims goes beyond simply attributing certain climatic conditions to ACC. Instead, the focus here is attributing certain harmful impacts to climate change. These are the focus of the PEA community when they suggest using this approach for justice or legal purposes (Allen, 2003, 2012; Allen et al., 2007; Thompson & Otto, 2015; Otto et al., 2017). Also, see the discussion below.

4 The storyline approach is not specifically designed for event attribution. Physical climate storylines have been defined as a self-consistent and plausible physical trajectories of the climate system, or a weather or climate event, on time scales from hours to multiple decades (Shepherd et al., 2018; Chen et al., 2021, Sect. 1.4.4.2) Such physical trajectories describe plausible future scenarios or past events and therefore can serve various purposes, from risk assessment of plausible climate change-related impacts to attribution of extreme weather events (see IPCC report, Ch 11, Sect. 2.3; Sillmann et al., 2021). Extreme event attribution relies on storylines of observed and counterfactual events. Moreover, note that the scientific community does not see physical climate storylines as a single concept (Jack et al., 2020).

5 We provide a longer discussion about these issues in Section 5 below. Moreover, we do not imply here to say that this is the only problem that PEA studies might face or that has been identified by other scientists. Others might include, for instance, the lack of a long enough observational record (van Oldenborgh et al., 2021). However, an extended discussion of these issues is beyond the scope of this paper.

6 Even more, scientists have started to work on how the storyline approach could be applied also to legal contexts. See (Lloyd & Shepherd, 2021).

7 Note that overstating and making false positives are not coextensive concepts. One can overstate an effect without necessarily claiming a false positive because no null hypothesis is tested, and not all false positives are overstatements because that depends on how the null hypothesis had been formulated. However, for the sake of the discussion, we stick to the concept of overstating because it captures the concerns behind this controversy in a more overarching way.

8 More details of the controversy have recently been reported in various pieces by Elisabeth Lloyd, Naomi Oreskes and Eric Winsberg (Lloyd & Oreskes, 2018, 2019; Winsberg et al., 2020).

9 We would like to thank an anonymous reviewer for highlighting this point.

10 Arguably, these do not exhaust all possible forms of overstatement. For instance, an attribution method could also overstate the effects of climate change by overstating decreases in likelihood or magnitude of an EWE. In this sense, an attribution method would affirm too much of a decrease in any of these parameters. However, this interpretation of overstatement is unlikely to capture the concern raised by the PEA community. Arguably, the reason is that overstating a decrease in likelihood or magnitude rarely implies overstating harmful impacts because these are mostly driven by increases (not decreases) in the likelihood and magnitude of EWE. For instance, cold spells are among the EWEs that have likely decreased in likelihood and magnitude due to ACC. However, decreases in cold spells rarely cause harm because societies tend to be adapted to average weather patterns. A decrease in the likelihood and magnitude of cold spells means that local weather moves closer to the average and thus stays more stable and within the limits of adaptation. We leave this interpretation of overstatement aside because the harmful impacts of climate change are the underlying focus of many attribution papers (Allen, 2003, 2012; Allen et al., 2007; Thompson & Otto, 2015; Otto et al., 2017).

11 O1 and O2 are closely connected but their relation is not necessarily of implication. First, overstatements of the magnitude of an EWE that only refer to how climatic conditions have been affected by ACC (O1) might not imply overstatements of the impacts caused by climate change. The reason is that an increase in the magnitude of an EWE (say, extreme temperature) might not be significant enough to overshoot thresholds associated with the occurrence of certain impacts, given that the relation is often not linear (see our example below). However, probabilistically, overstatements in sense O1 lead to overstatements in sense O2. Second, O2 often implies O1 because, in this context, overstatements of the impacts attributable to ACC (O2) are derived precisely from overstatements of the magnitude of the EWE because of ACC (O1). However, it is also possible that the severity of an event is not overstated but that the impacts resulting from the occurrence of an event of this severity are overstated. For instance, one might accurately describe the effects of ACC on rainfall (event), but overstate the impacts of rainfall on landslides, particularly if one misrepresents the effects of the interplay between rainfall and other co-founding factors (such as soil moisture) on the severity and occurrence of landslides (Perkins-Kirkpatrick et al., 2022).

12 Such studies derive so-called response surfaces or impact functions (e.g. Prudhomme et al., 2010), which quantify the response of an impact (e.g., river runoff) to changes in key drivers.

13 Admittedly, such a threshold would not prevent all possible overstatements. In cases in which the storyline overstates how much ACC increased the severity of events whose magnitude is situated above the threshold to attributing certain harmful effects to ACC, this methodology would indeed overstate the effects of ACC in sense O2. However, this threshold would at least limit the risk of overstating the impacts of ACC. We thank the editor for pointing out this issue. Furthermore, it is worth noting that the combination of PEA with a 0.5 FAR threshold is also not free from threshold-related problems since it delivers the same attribution statements for, e.g., 0.6 FAR events and 1.0 FAR events. That is, the (arguably important) differences in changes on the probability of occurrence due to ACC are not reflected in the attribution statements.

14 We are aware that selecting the full ensemble across all groups will avoid overstatements of ACC because opposing changes may result in an inconclusive statement about the influence of ACC (‘we don’t know yet’) (Shepherd, 2016). Probably, this is the reason why it has been argued that the probabilistic approach tends to understate the effects of ACC (Winsberg et al., 2020; Lloyd & Oreskes, 2019). However, here we want to highlight that the (not uncommon) selection of a small number of models can also yield overstatements, something that has been underemphasized in the literature.

15 In general, model ensembles are not designed to fully sample uncertainties but based on availability. They are referred to as ensembles of opportunity and typically underestimate true uncertainties (Tebaldi & Knutti, 2007).

16 Selecting models to reduce climate change uncertainties is a challenging topic of active research (Eyring et al., 2019) but essentially unresolved. Thus, the IPCC states ‘there is high confidence that ensembles for regional climate projections should be selected such that models unrealistically simulating processes relevant for a given application are discarded, but at the same time, the chosen ensemble spans an appropriate range of projection uncertainties.’ (Doblas-Reyes et al., 2021).

17 Although we are not aware of case studies showing how the use of only a few models yields overstatements, our argument hints at this possibility, for the reasons provided in the main text. Partly in response to this problem, the approach to estimating the FAR has been generalized to be able to make use of existing multi-model ensembles (WWA, 2021; van Oldenborgh et al., 2021), although the recommendations of the WWA for model selection should be amended by a criterion that includes representing the full spread in relevant dynamic aspects. However, in practice, many studies are still based on a small number of models. Some examples include (Otto et al., 2018; Kirchmeier-Young et al., 2017).

18 Note that presence/absence of rain is determined by the weather type (meteorological drought) and thus by the dynamics. The intensity or severity of the rain would be related to the thermodynamics (Clausius Clapeyron).

19 As a matter of example, Stott et al. (2004) paper seemingly aims to analyze the ‘human contribution to the European heatwave of 2003’ (as the title goes). Hence, the initial and motivating attribution question refers to the 2003 event, which was caused by the interplay of various dynamic and thermodynamic factors. However, the results of the PEA study only show the influence of ACC on ‘unusually high mean summer temperatures.’ Our point here is that if the results of this PEA study, for instance, are interpreted as providing an answer to the initial attribution question concerning the multi-dimensional 2003 event (which would not be surprising given that the results are provided as an answer to such a question) such an interpretation might lead to overstatements. We are aware that this is not an inherent problem of PEA studies, but rather on how they are received by stakeholders. However, we believe that the risk of misinterpretation and thus of overstatements is real and it deserves attention, even if it is only to avoid them.

20 Some researchers have developed attribution approaches for compound events (Mazdiyasni et al., 2019; Kiriliouk & Naveau, 2020), which can account for the dynamic aspects (and other variables such as precipitation or wind, Zscheischler et al., 2020), but these approaches are not widely used and still suffer from the lack of robustness in projecting dynamical changes.

21 Note that this argument holds for the mid-latitudes. In the subtropics, also dynamic changes may be very robust because of their direct link to the Hadley cell (Cresswell-Clay et al., 2022).

22 Note that the use of proxy-definitions also challenges the idea that the probabilistic approach treats an EWE as a ‘single, self-reinforcing and indivisible whole’ (Allen, 2012, 13), see Section 2.
